# High-Solids, Solvent-Free Modification of Engineered Polysaccharides

**DOI:** 10.3390/molecules26134058

**Published:** 2021-07-02

**Authors:** Athanasios Porfyris, Constantine D. Papaspyrides, Natnael Behabtu, Cristian Lenges, Alexander Kopatsis

**Affiliations:** 1 Laboratory of Polymer Technology, School of Chemical Engineering, National Technical University of Athens (NTUA), Zographou Campus, 15780 Athens, Greece; 2 IFF Nutrition & Biosciences, Willem Einthovenstraat 4, 2342 BG Oegstgeest, The Netherlands; Natnael.Behabtu@iff.com; 3 IFF Health & Biosciences, Engineered Polysaccharide Venture, 200 Powder Mill Road E353, Wilmington, DE 19803, USA; Christian.P.Lenges@iff.com (C.L.); Alexander.D.Kopatsis@iff.com (A.K.)

**Keywords:** polysaccharides modification, α-(1,3) glucan, acylation

## Abstract

The nature-identical engineered polysaccharide α-(1,3) glucan, produced by the enzymatic polymerization of sucrose, was chemically modified by acylation with succinic anhydride. This modification reaction was initially performed at the micro scale in a TGA reactor to access a range of reaction conditions and to study the mechanism of the reaction. Subsequently, the best performing conditions were reproduced at the larger laboratory scale. The reaction products were characterized via coupled TGA/DSC analysis, FT-IR spectroscopy, solution viscosity and pH determination. The acylation path resulted in partially modifying the polysaccharide by altering its behavior in terms of thermal properties and solubility. The acylation in a solvent-free approach was found promising for the development of novel, potentially melt-processable and fully bio-based and biodegradable ester compounds.

## 1. Introduction

Polysaccharides are abundant, sustainable and versatile biopolymers with a long tradition in industrial applications [1]. The conversion of cellulose to cellulose-esters and ethers was pioneered and commercialized in the early parts of the last century, and these materials have also been in use as early thermoplastics. However, key limitations remain to the currently available polysaccharide materials, based on required processing conditions, high reinvestment economics and in market applications potential. Traditionally, large scale naturally sourced polysaccharides (e.g., cellulose, starch) are extracted and refined from plants to constitute the most abundant polymer resource, which are considered as sustainable renewable resources [2,3,4]. Chemical modification to enable a wide series of new products and materials provides an attractive development objective. However, there are also some disadvantages, since most natural polysaccharides are extracted as mixtures, e.g., starch consisting of amylopectin and amylose in a typical ratio of 3:1 with a typical and specific glycosidyl-linkage type. Control of the desired material properties can be affected through control of linkage isomers, polymer molecular weight and weight dispersity [5,6,7,8].

**Scheme 1:**
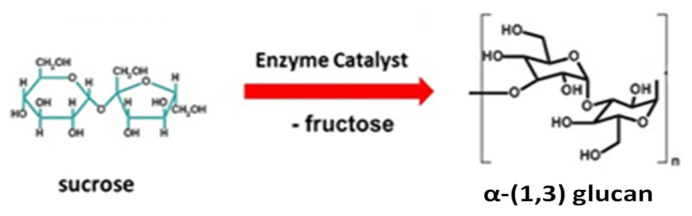
Synthesis path for engineered polysaccharide [9].

Recently, the enzymatic polymerization of sucrose to generate nature-identical polysaccharides of defined and desirable properties has been under development [9,10]. Accordingly, specific linkage control can tune the properties of the material. As an example, the polysaccharide described in this paper consists of exclusively α-(1,3) linked glucose molecules. The target molecular weight can be tuned to a wide range of 50 to 150 kg/mol. This polysaccharide shows a semicrystalline structure with a high crystallinity index, usually higher than 50%. As is typical for highly aggregated polysaccharide materials, this material is not directly melt-processable, and decomposition occurs before any viable flow is observable. Also, the parent alpha-1,3-polysaccharide does not dissolve in water, and for many applications, water solubility is desired. (Scheme 1).

The invention of cellophane in 1912 [11], i.e., a transparent film used in packaging applications that is derived from cellulose and carbon disulfide (CS_2_) via a complex solution-based process, proves the historic use of refined cellulosic materials in industrial applications. The particular film is still used today in the packaging sector, but it is constantly being replaced by alternative, lower-cost, melt-processable materials, which are meanwhile manufactured by lower-emission processes [6]. For example, thermoplastic starch is used for the manufacture of packaging films and drug capsules [12,13,14], while cellulose ester derivatives are used in membranes, films and fibers [7,15]. Nevertheless, the properties of these materials are dominated by the fixed structure of the initial natural polysaccharide feedstock, and any adjustments of that underlying structure in a systematic approach have not been possible.

On the contrary, the α-(1,3) glucan system derived from enzymatic polymerization, because of its adjustable polymer architecture, can result in product features with unique properties in comparison to its extracted counterparts [9]. Meanwhile, the readily fungible and renewable agricultural feedstock used for the enzymatic polymerization allows for an environmentally sustainable bioprocess, which results in a fully bio-based, readily biodegradable polymer that constitutes a sustainable and scalable material source. The use of the polysaccharide α-(1,3) glucan has been reported for a range of applications, for example, as reinforcing filler for polypropylene (PP), polyethylene (PE) and polylactic acid (PLA) composites to enable the enhancement of tensile strength and thermal stability while not impacting the required aesthetic properties of such composites [9,16,17].

In this paper, focus is given on the development of innovative glucan ester derivatives, with potential applications in the packaging sector. In fact, since this type of polysaccharide has not been available in large quantities for industrial assessment, limited literature data regarding the chemical modification exists. Nevertheless, similar pathways to the modification of traditional extracted polysaccharides can be followed, e.g., esterification of cellulose with carboxylic acids or acylation with cyclic dianhydrides, since glucans constitute compounds of similar general structure [7,18].

Regarding the direct esterification of cellulose with carboxylic acids, for example, long-chain fatty acids (8-16 carbon atoms) are reacted with the free hydroxyl groups so that polysaccharide esters are formed with a mixed degree of substitution, which show much lower crystallinity, and eventually a melt-processable composition is achieved [7]. Such reactions have been described using a solution system, e.g., dimethylacetamide with dissolved lithium chloride (DMAc/LiCl), because the intense hydrogen bonding of neighboring hydroxyl groups requires disruption, rendering these groups much more reactive. The challenge herein is to “activate” the low reactivity fatty acids by a proper catalyst, e.g., *p*-toluenesulfonyl chloride (Tos-Cl), so that esterification can proceed. According to literature, a degree of substitution (DS) of up to 2.5 can be achieved at 80 °C and 24 h of reaction time [7,19,20].

Alternatively, esterification of glucan with short-chain carboxylic acids (2–8 carbon atoms) is feasible, and the respective esters can be formed via heterogeneous or homogenous processes [7,21,22]. Regarding the heterogeneous approach, glucan can be mixed with the equimolar ratio of the carboxylic acid and trifluoroacetic anhydride (TFAA) at 50 °C, and the reaction for achieving DS = 3 requires 3 h. Nevertheless, the disadvantage of this process is the intense chain scission of the polysaccharide due to the acidic conditions [20,21]. On the other hand, in the homogenous method, the glucan is added in DMAc/LiCl at 60 °C until the solution turns clear. Subsequently, the desired short chain acid anhydride is added, e.g., butyric anhydride, hexanoic anhydride or caprylic anhydride along with pyridine as catalyst [22]. This reaction path involves only monofunctional anhydrides. Full substitution of the hydroxyl groups, i.e., DS = 3, can be achieved only after 96 h of reaction. By following this reaction path, the degradation of the polysaccharide is avoided, but the reaction time is very long, and the use of a solvent system at low glucan solid load renders the potential process industrially not viable. Both aforementioned processes can result in melt-processable glucan esters with melting points ranging from 142 to 339 °C [22].

Turning to the case of modifying natural polysaccharides, e.g., cellulose, by acylation with dianhydrides, a more direct reaction is anticipated due to the higher reactivity of these anhydrides, such as maleic, succinic and phthalic [7,18]. Here, the anhydride group reacts with the hydroxyl groups of the polysaccharide and is added on the polymer backbone (no byproduct involved), resulting in the formation of the polysaccharide ester (ring opening of the anhydride) [7,18,23]. In this reaction, the hydroxyl groups are substituted with carboxyl ones, offering additional options to adjust the hydrophilic character of the product. The reaction temperature is determined by the melting point of the used anhydride, and the anticipated reaction time required is expected to be short, e.g., 1 h [18]. Typically, this type of reaction is carried out in a solvent medium. For example, the reaction of cellulose with succinic anhydride can be carried out in DMSO at 80–120 °C for up to 6 h resulting in DS ranging from 1.5 to 2.2, but the received substituted product is not melt-processable [19,23]. Alternatively, reactive extrusion has also been used for the acylation of cellulose or starch, where high temperature along with shear stress result in partial substitution of the used polysaccharide [24,25].

The objective of this work has been to chemically modify α-(1,3) glucan via ring-opening acylation with succinic anhydride (SA) so as to obtain the respective polysaccharide ester. Such a product composition may be of interest for a range of applications, for example in the area of adhesives, coatings or packaging applications. The challenge that arises is to carry out the reaction only by direct contact of the reactants and to obtain the desired DS value so that the final product may further processed (e.g., by melt-processing or dissolution). The experimental work was initially carried out at the micro scale so as to scout the proper reaction conditions [26,27,28]. Then, laboratory scale runs in different assemblies were performed so as to confirm the findings from the micro scale. This approach is modeled after the fundamentals of the solid state polycondensation (SSP) of polyamide salts, and lessons from this research field have been adapted in this work [27,28,29,30]. Thus, a TGA aluminum crucible was used as a micro-scale reactor. The starting material was kept at a given temperature and gas flow, and the reaction progress was assessed over time by monitoring the mass loss of the formed byproduct. This technique excludes any mass and/or heat transfer effects, thus revealing the inherent kinetics of the reaction. This work describes a novel attempt to prepare glucan derivatives with utility in a series of applications, providing sustainable, renewable and biodegradable alternatives.

## 2. Results and Discussion

### 2.1. Characterization of α-(1,3) Glucan

**Figure 1:**
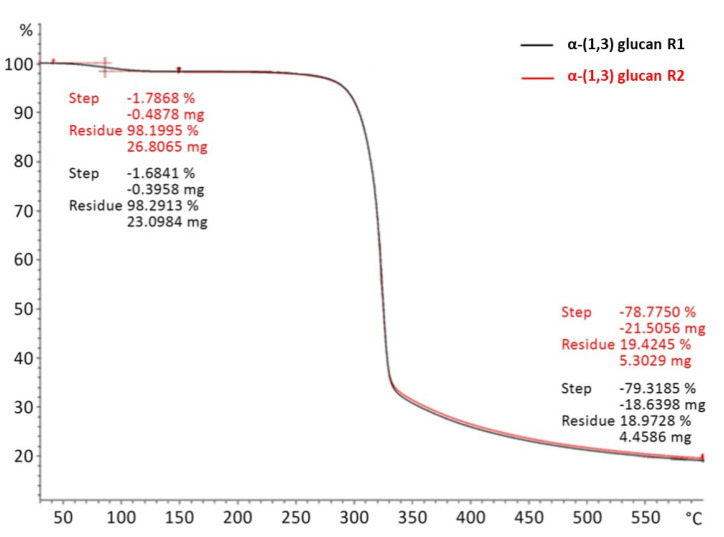
TGA runs on the neat α-(1,3) glucan.

The neat material, in the form of fine and free flowing powder, was characterized initially by TGA/DSC analysis, FT-IR spectroscopy and solution viscosity. Accordingly, the material prior to any characterization was intensively dried under 70 °C overnight followed by 3 h at 100 °C under 400 mbar vacuum so as to sweep off the adsorbed water. This is of high importance since the high concentration of hydroxyl groups renders the material highly hydrophilic. In Figure 1, the TGA curves of the neat material are presented, where a T_d_ of 331 °C and a relatively high char residue of ca. 19 wt.% were determined. A residual water content of ca. 1.8 wt.% was also observed, although the material was thoroughly dried. This underlines the intense hydrophilicity of the material.

**Figure 2:**
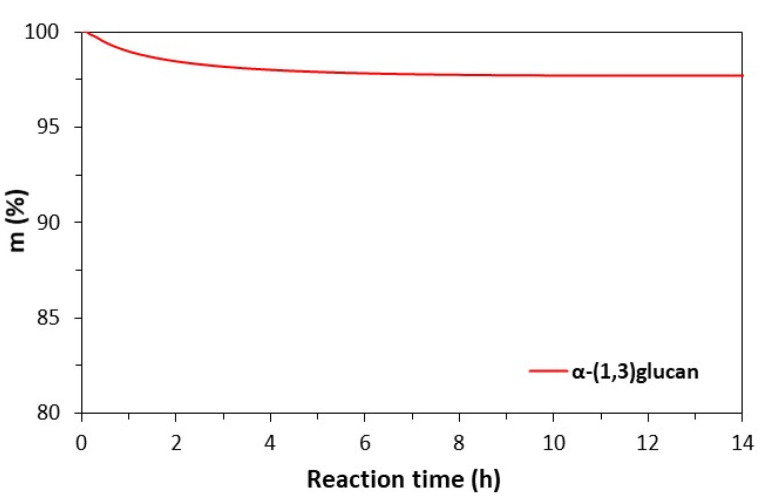
TGA run on the neat α-(1,3) glucan at 130 °C for 14 h.

TGA runs were carried out on neat glucan for long times and at high temperatures. In Figure 2, a TGA curve at 130 °C for 14 h is shown. This profile was applied for all acylation reactions. The data indicate the mass stability of the neat glucan under these conditions; however, this not being the case for the molecular size is discussed below in viscosity runs.

Turning to the solution viscosity of the neat glucan, an intrinsic viscosity ([*η*]) value of 2.7 dL/g was determined. Moreover, the typical degree of polymerization for the engineered polysaccharide consists of ca. 800 repeating glucose units (source: DuPont Nutrition and Biosciences). Since the molecular weight of each repeat unit is 162 g/mol, the material shows a MW of ca. 129.6 kg/mol. Nevertheless, it should be mentioned that when the material is subjected to ten (10) repetitive drying cycles so as to maintain the humidity level at ca. 2 wt.%, the [η] value decreased to 1.53 dL/g. This proves the susceptibility of this particular polysaccharide to thermal degradation.

**Figure 3:**
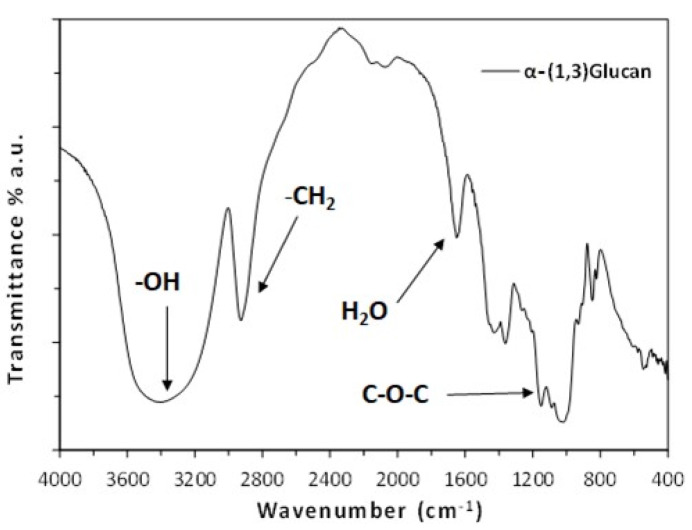
FT-IR spectrum of dried neat glucan.

Finally, an FT-IR spectrum of dried neat glucan is shown in Figure 3. Similarities with other polysaccharides, such as cellulose or starch, were observed [3]. Regarding the peak assignment, the hydroxyl groups (stretch vibrations O-H) are presented at ca. 3300 cm^−1^, the symmetric and anti-symmetric stretch vibrations of the -CH_2_ groups are observed at ca. 2900 cm^−1^ and at 1125 cm^−1^, the antisymmetric stretch of the C-O-C bond, i.e., of the glycosidic linkage is observed. Finally, at 1635 cm^−1^, the adsorbed water is shown [3].

### 2.2. Acylation of α-(1,3) Glucan with Succinic Anhydride

#### 2.2.1. Acylation Reactions at the Micro Scale

Modification reactions were initially carried out in the micro scale of the TGA reactor. The equimolar calculations for target DS = 1 are shown in Table 1, and an optional addition of 10 wt.% NaPO_2_H_2_·H_2_O as a catalyst is included [31]. Here, a target DS value of 1 was selected on purpose to avoid potential undesirable intense cross-linking of the polysaccharide. Regarding the calculation of the reacting groups in Table 1, each glucose ring has three free hydroxyl groups (-OH), or 3 eq, and if the 800 repeating glucose rings are taken into account, then the total [-OH] is 2.400 eq. On the other hand, the molecular weight of α-(1,3) glucan is 129.6 kg/mol, which means that 129.6 kg of the polysaccharide have 2400 eq of [-OH]. If this is reduced to the 1 kg scale, then it yields 18.519 eq/kg or 18,519 meq/kg. The reacting groups of SA were similarly calculated, nevertheless only one group reacted the hydroxyl groups of the polysaccharide.

**Figure 4:**
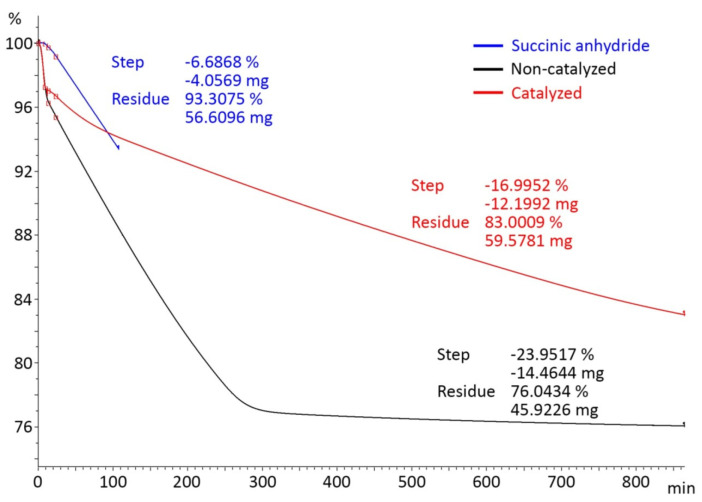
Acylation of α-(1,3) glucan with succinic anhydride in the TGA micro reactor.

All runs were carried out above the melting point of the SA (122 °C) at 130 °C for 14 h. The pertinent acylation reaction does not involve a byproduct, and therefore no mass loss was anticipated. However, the mass loss curves quoted in Figure 4 indicate that the neat anhydride (blue line) was volatile at this temperature and the extrapolated loss at 14 h would be ca. 52 wt.%. Turning to the noncatalyzed case (black line), a mass loss of 23.95 wt.% was recorded due to SA sublimation, also reaching a plateau.

If we reduce our calculations to 100 g of GL, then from 61.80 g of SA loaded in the reactor only 32.14 g would escape sublimation. Furthermore, if all remaining 29.66 g had reacted, a DS value of ca. 0.48 would be obtained. This is of course based on the aforementioned extrapolated SA loss of ca. 52 wt.% at 14 h and also on the assumption that all SA remained would be reacted and not entrapped into the modified glucan. In other words, this value might be overestimated. Coupled DSC/TGA runs on the TGA products, as below, confirm this approach.

On the contrary, regarding the catalytic experiment with NaPO_2_H_2_·H_2_O (red line), a different reaction mechanism was anticipated. Therein the total mass loss was 16.995 wt.%, or 29.044 wt.% less than in the uncatalyzed case. This might indicate that since less SA was sublimed, more SA would be available for reaction. Thus, it seems that the catalyst contributed in “protecting” the SA from sublimation, and therefore the acylation performance was improved in terms of the DS value attained.

This aforementioned mechanism was confirmed via the larger scale experiments presented below. However, the obvious change of the reaction mechanism does not permit a safe evaluation of the DS value attained here. Last but not least, the acylation reaction was not completed within the 14 h time limit chosen.

**Figure 5:**
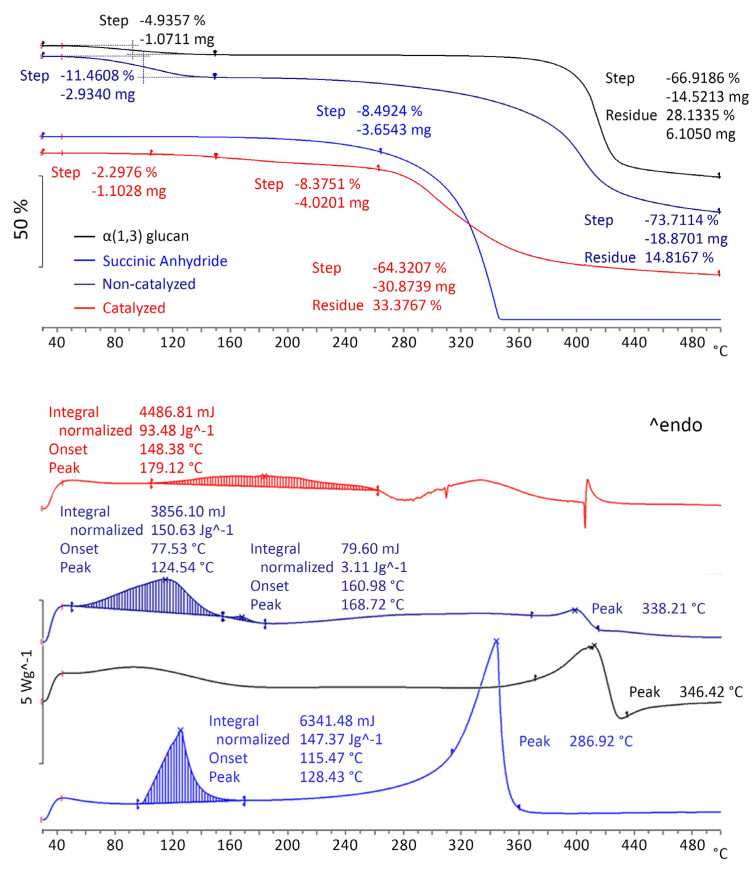
Coupled TGA/DSC runs on products derived from the modification of α-(1,3) glucan with SA in micro scale.

Coupled TGA/DSC analysis was further performed on the as-received products of the micro-scale runs (Figure 5; neat reactants are first shown for comparison). Accordingly, α-(1,3) glucan (black line/DSC) shows no melting peak except a very broad one assigned to the inherent humidity, while the neat succinic anhydride (blue line) shows an intense melting endotherm at 128.43 °C.

Regarding the acylation runs, the noncatalyzed one (dark blue line) resulted in an intense endotherm at 124.54 °C assigned to melting of unreacted SA. This peak is followed by a small endotherm at 168.72 °C, which should correspond to melting of the substituted glucan formed. Therefore, it is anticipated that the reaction partially occurred, but the as-received product contained unreacted SA. This confirms the suspected overestimation of the DS value discussed earlier (0.48).

On the contrary, in the case of the catalyzed experiment (red line), no endotherm assigned to SA melting was observed as before. Instead, a very broad endotherm at 179.12 °C was recorded. It can be concluded that partial acylation was again achieved, but with no entrapment of unreacted SA in the as-received product.

#### 2.2.2. Acylation Reactions at Laboratory Scale

The aforementioned micro scale data were reproduced at the laboratory scale so as to verify the findings. Both catalyzed and uncatalyzed experiments were performed again at 130 °C for 14 h. The products, as received after the reaction, were washed thoroughly with water so as to remove any unreacted SA (and the catalyst employed) and then dried in vacuum and weighed. The initial mass compositions used are quoted in Table 2 together with pertinent data after the reaction.

Table 2 reveals first that in both cases (noncatalyzed and catalyzed), the as-received products contained remarkable quantities of unreacted SA. However, the latter seemed to decrease very significantly in the presence of the catalyst: 2.4 g/3.8 g versus 3.3–1.62 g/6.23 g or 63.16% versus 26.97%. Thus, the data are aligned very well with the TGA runs in Figure 4. Furthermore, the catalyst nearly doubled the attained DS value in comparison with the noncatalyzed run (0.70 versus 0.37). Last but not least, the latter value of 0.37 verifies, at least qualitatively, the overestimation of the DS value of 0.48, discussed already in the TGA runs.

**Figure 6:**
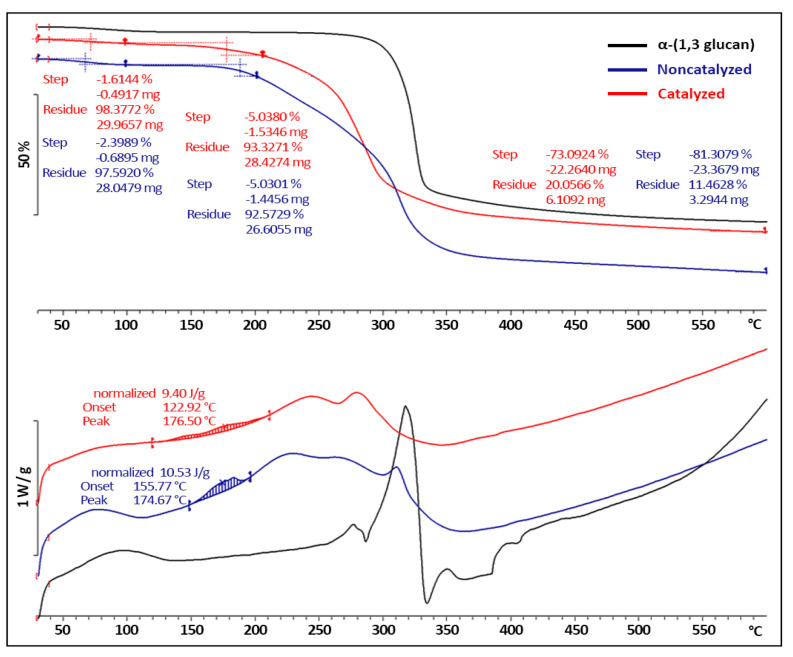
Coupled TGA/DSC runs on products derived from the modification of α-(1,3) glucan with SA in laboratory scale.

Turning to the coupled TGA/DSC analysis on the washed products of the laboratory-scale runs (Figure 6), neat glucan is first shown for comparison (black line). It is obvious that apart from the humidity endotherm at ca. 100 °C, no other endotherm is observed up to 331 °C, where the decomposition of the polysaccharide occurs. On the contrary, both acylated products exhibited melting endotherms in a temperature range between ca. 120 and 200 °C. More specifically, the noncatalyzed grade (dark blue line) shows a melting peak at 174.67 °C, while the catalyst-containing one (red line) shows a broader endotherm at 176.5 °C. Both cases are completely in harmony with the findings from the TGA scale (Figure 5), verifying once again successful acylation. Nevertheless, the thermal stability of these substituted materials is worse of that of neat glucan, indicating some chain-scission, again due to the reaction conditions (long time at a high temperature and acidic environment).

**Figure 7:**
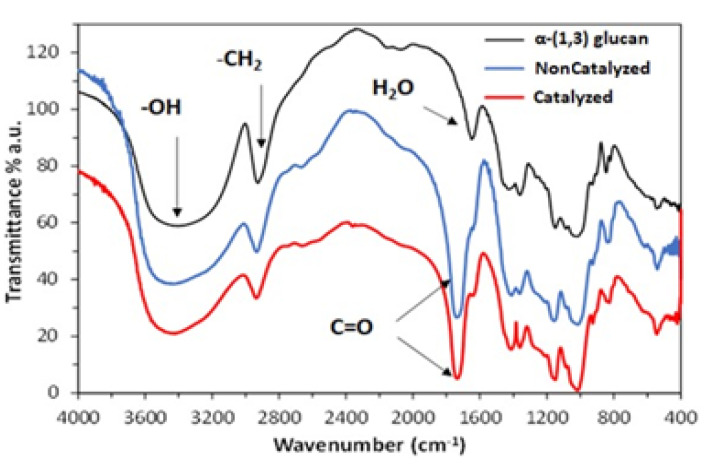
FT-IR spectra of the acylated glucan grades and comparison with the neat grade.

In order to further verify the new structure formed, the FT-IR spectra of the products were compared to that of the initial glucan (Figure 7). The new peak at ca. 1730 cm^−1^ corresponds to the stretching vibration of the C=O bond, which verifies the successful acylation in both cases, while the peak at 3370 cm^−1^ is weaker compared to the neat grade, verifying again the decrease of the hydroxyl groups in the polysaccharide lattice.

Turning to pH characterization, the pH value of the neat α-(1,3) glucan was compared to that of the modified grades. Glucan provides a basic pH of 7.7, compared to the slightly acidic 4.4 of both the noncatalyzed and catalyzed grades. Obviously, this is due to the dissociation of the new carboxyl groups formed.

**Figure 8:**
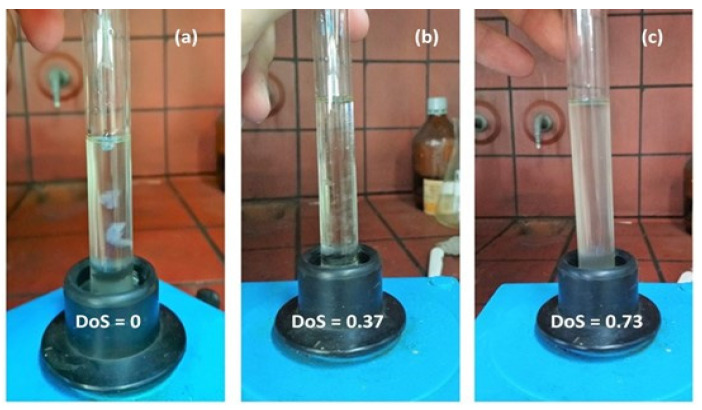
Solubility trials in DMSO. (**a**) α-(1,3) glucan; (**b**) noncatalyzed; (**c**) catalyzed.

Last but not least, it is worthwhile mentioning that solution viscosity measurements were not possible since the products remained insoluble in DSMO/LiCl, once again proving acylation. In particular, solubility trials with pure DMSO were attempted (Figure 8). In these trials, neat glucan was not dissolved but became aggregated and swollen (Figure 8a), while the noncatalyzed sample (Figure 8b) exhibited a different behavior of separate small particles in suspension. Finally, the catalyzed sample (Figure 8c) was almost soluble in DMSO, proving the successful acylation by completely altering the solubility behavior.

## 3. Materials and Methods

The polysaccharide α-(1,3) glucan was used as received (DuPont, now IFF, Wilmington, DE, USA). Succinic anhydride was received from Alfa Aesar. As catalyst, the monohydrate of sodium hypophosphite (NaPO_2_H_2_·H_2_O) was employed, received from Sigma-Aldrich (St. Louis, MO. USA).

All modifications of α-(1,3) glucan were first investigated at the micro scale in a Mettler Toledo TGA/DSC 1 HT instrument using 100 μL aluminum crucibles [22,23,25]. Approximately 50 mg of the premixed reactants (glucan, modifier and catalyst if any) were placed in the crucible, which was then sealed with a lid having a hole on the top of 2 mm diameter. The samples were inserted into the TGA chamber at 30°C (*T*_0_), preheated to 120 °C (*T*_1_) at a rate of 20 °C/min, left at *T*_1_ for 5 min and further heated up to the nominal reaction temperature (*T*) at a rate of 1 °C/min, followed by the final isothermal step at *T* for periods ranging from 14 to 20 h (t). Nitrogen was used as the inert gas at a constant flow rate of 10 mL min^−1^. After the isothermal step, the material was cooled to room temperature.

The reactions with succinic anhydride (SA) at laboratory scale were performed in a rotary evaporator apparatus (BUCHI Rotavapor R210). The preweighed amounts of the reactants, i.e., α-(1,3) glucan, SA and optional catalyst, were added together into the 500 mL round bottom flask, which was then mounted to the rotary evaporator. The flask was then immersed in the oil bath of the apparatus to be heated at 130 °C for 14 h so as to mimic the TGA runs. The rotation of the apparatus was adjusted so that the reaction mass always remained well mixed.

Coupled TGA/DSC analysis was performed on all samples in a Mettler Toledo TGA/DSC HT1 instrument. Samples were heated from 30 to 600 °C at a heating rate of 10 °C/min with simultaneous nitrogen flow controlled at 10 mL/min. Approximately 8-10 mg of each sample was placed in an alumina crucible and then in the TGA chamber. From the received curves, the following values were determined: temperature at 5 wt.% loss (*T*_d,5%_), maximum decomposition temperature (*T*_d_), melting point (*T*_m_) and melting enthalpy (Δ*H*_m_).

FT-IR spectra of the herein-tested samples were received in a Bruker Tensor 27 spectrometer. For each region, a series of spectra were recorded, and every spectrum consisted of 32 coadded spectra from 4000 to 400 cm^−1^ at a resolution of 4 cm^−1^. Pastilles of each sample with KBr were prepared, and air was received as background.

Solution viscosity measurements were performed in order to follow any variation in the molecular weight after a chemical modification. The measurements were performed on fully dissolved glucan solutions of 0.5 wt.% in DMSO/3% LiCl at 40 °C in a temperature-controlled water bath by the use of a Cannon Fenske type viscometer (K = 0.2308 mm^2^/s). The outflow time(s) was measured in triplicate, and the intrinsic viscosity value [*η*] was determined according to equation 1, where C is the solution concentration (g dL^−1^), *η*_*sp*_ the specific viscosity of the solution [32].

(1) $$\left[\eta \right]=\frac{\sqrt{1+1.5\eta_{sp\;}}-1}{0.75C}$$

One percent by weight aqueous solution or slurry of a sample was prepared, and the pH was determined with a Hanna Instruments pH211 instrument. The instrument was calibrated with buffer solutions of pH 4, 7 and 9.

## 4. Conclusions

In this work, the chemical modification of α-1,3 glucan was carried out via acylation with succinic anhydride in the absence of any solvents. On the micro scale (TGA) and in laboratory-scale conditions, a DS of up to 0.70 was achieved. The successful acylation was verified by increase in the mass yield of the final product, by coupled DSC/TGA scans, by FT-IR spectroscopy and by solubility trials in DMSO. All these characterization techniques suggested the partial substitution of the polysaccharide and the formation of the derivatized material. This route may also enable direct, salt-free access to a polyanionic but biodegradable polysaccharide-based system without the use of any solvents (high-solids and solvent-free technique). These remarks render the herein applied technique very promising for the production of novel, fully bio-based and biodegradable esters of α-(1,3) glucan, arising as sustainable alternatives to traditional petrochemical polymers in the field of packaging. Last but not least, it is shown in this example how the utilization of a micro-scale screening method, such as the TGA reactor used here, can provide the accuracy for predicting reaction profiles in the laboratory scale, even 5000 times larger.

## Figures and Tables

**Table 1:** Equimolar calculations for modification of α-(1,3) glucan with succinic anhydride.

Reactants	MW (g/mol)	Reacting Groups (meq/kg)	Mass Ratio (GL:SA)
α-(1,3) glucan	129,600	18,519	-
succinic anhydride	100.1	19,980/2 = 9990	1:0.618 ^1^

^1^ Target DS = 1.

**Table 2:** Reaction data for laboratory-scale modification of the α-(1,3) glucan with succinic anhydride.

**Initial Composition (Mass Ratio GL:SA = 1:0.62 for Target DS = 1)**				
**Experiments**	**Glucan Neat (g)**	**SA (g)**	**NaPO_2_H_2_^.^H_2_O (g)**	
Noncatalyzed	6.2	3.8	0	
Catalyzed (10 wt.% NaPO_2_H_2_^.^H_2_O)	10.54	6.23	1.62	
**Received Products after Reaction**				
	**Modified Glucan (g)**	**Received SA (g)**	**DS**	**[*η*] (dL/g)**
Noncatalyzed	7.6	2.4	0.37	Insoluble
Catalyzed (10 wt.% NaPO_2_H_2_^.^H_2_O)	15.1	3.3	0.7	Insoluble

## Sample Availability

Samples of the modified compounds are available from the authors.
